# A Chirped Characteristic-Tunable Terahertz Source for Terahertz Sensing

**DOI:** 10.3390/s24165419

**Published:** 2024-08-22

**Authors:** Feilong Gao, Mingzhe Jiang, Shaodong Hou

**Affiliations:** 1 School of Physics Science and Information Technology, Liaocheng University, Liaocheng 252059, China; gaofeilong@lcu.edu.cn (F.G.); 2020203519@stu.lcu.edu.cn (M.J.); 2 Changzhou Inno Machining Ltd., Changzhou 213164, China

**Keywords:** terahertz radiation, chirped beam, optical rectification, terahertz sensing

## Abstract

In broadband terahertz waves generated by femtosecond lasers, spatial chirp will be simultaneously produced with the introduction of angular dispersion. The chirp characteristics of the terahertz wave will directly affect the frequency response, bandwidth response, and intensity response of the terahertz sensor. To enhance the capability of terahertz sensors, it is necessary to control and improve the chirped characteristics of broadband terahertz sources. We generate a chirped terahertz wave via optical rectification in a LiNbO_3_ prism using the technique of pulse front tilt. The effect of the pump-beam spot size on THz generation is systematically studied. The pump’s spot size is manipulated using a telescope system. With a pump spot diameter of 1.8 mm, the scanning spectrum of the THz pulse is narrower and is divided into multiple distinct peaks. In contrast, using a pump spot diameter of 3.7 mm leads to increased efficiency in the generation of THz pulses. Also, we investigate the underlying properties governing the generation of chirped terahertz pulses using varying pump pulse spot diameters.

## 1. Introduction

The terahertz (THz) wave is an electromagnetic wave within the frequency range of 0.1 to 10 THz. Due to its unique position in the electromagnetic spectrum, THz waves exhibit distinct properties and are utilized in various fields, such as molecular dynamics, imaging, quality monitoring, security inspection, and communication [1,2,3,4,5].

Terahertz waves are also suitable for sensing applications, serving to complement or enhance conventional sensing technologies. The fundamental concept of THz sensing involves deriving information from the interaction between THz waves and objects. When the target object is irradiated with THz waves, interactions occur with its molecules, atoms, or electrons, resulting in unique response signals. These signals contain specific information about the internal structure, composition, thickness, and various other characteristics of the object. Through the detection and analysis of these signals, THz sensors can achieve non-contact and non-destructive testing of the subject.

Compared to traditional sensing technologies, THz sensing technology presents notable advantages. Firstly, THz waves have the ability to permeate various non-metallic and non-polar materials, such as plastics, ceramics, and paper, without inducing damage, thus enabling non-destructive testing. Secondly, THz sensing technology offers high temporal and spatial resolution, facilitating the identification of complex structures and transient alterations within materials. Furthermore, THz waves exhibit relatively low energy levels, making them less harmful to human health. These advantages have led to the continuous expansion of THz sensing technology applications. In the field of biomedicine, researchers employ terahertz sensing technology for tissue imaging, drug analysis, and disease diagnosis [6,7,8]. By observing the reaction of biological tissues to terahertz waves, a deeper understanding of their structure and functional condition can be achieved. Additionally, this technology aids in the identification and quantitative analysis of drug molecules [9]. In industrial detection applications where precision and non-contact measurement play a critical role in enhancing product quality and production efficiency, terahertz sensing technology has emerged as a valuable tool [10,11]. For example, in the electronics manufacturing sector, terahertz sensors can detect small defects or foreign objects on circuit boards [12], while in the food packaging industry, these sensors can be utilized for thickness measurements and evaluations of sealing integrity [13].

The sensitivity of THz sensors is influenced by various factors, with the properties of the THz source being crucial. The chirp characteristics of the THz source significantly impact sensor sensitivity. The stability of the THz source’s frequency directly affects resolution and measurement precision. Frequency fluctuations can increase noise in the sensor’s output, reducing sensitivity. The output power of the THz source determines the minimum detectable signal strength, with higher power improving sensitivity. A broader bandwidth enhances the sensor’s capability to identify intricate samples. Optimizing the THz source’s chirp characteristics can improve sensor performance for precision measurement and detection applications. Optical rectification in nonlinear crystals is employed as a common method for generating THz pulses. Efficient THz generation through optical rectification requires phase matching between the pump laser and the THz wave, meaning that the group velocity of the optical pump pulse and the phase velocity of the THz radiation must be matched [14,15]. Pulse front tilt (PFT) of the pump laser pulse is a valuable technique for achieving phase matching [16,17]. A PFT technology without a grating was first reported by Bowlan and Trebino [18]. A reflective stair-step device was also reported by Benjamin K et al. to generate THz waves [19]. The generation of THz waves in a LiNbO_3_ (LN) prism using a digital micromirror device has also been reported, thereby replacing the grating-based approach [20]. The pump beam used in the aforementioned PFT methods was discontinuous, resulting in a division of the generated THz radiation.

Grating can be used as a simple, effective, and low-cost device for PFT. In particular, the use of grating enables the implementation of continuous PFT. The principle of PFT by grating is based on angular dispersion. However, spatial chirp will be induced in the laser beam. A THz wave generated by a laser with spatial chirp appears significantly different compared to that generated by a laser without spatial chirp. A simple method to demonstrate the chirped property of such a THz wave is to utilize a pump beam with varying spot sizes. When a pump beam with a large spot size is incident on the grating, the separated frequency diffraction area will be large and superimposed, leading to a less pronounced chirped property of the pump beam. However, the chirped property becomes more evident when using a pump beam with a smaller spot size. The chirped property of the output THz wave will exhibit significant differences when different chirped lasers are employed [21].

The performance of terahertz sensors is highly dependent on the chirped characteristics of the terahertz source. To enhance the capability of terahertz sensors, it is necessary to control and improve the chirped characteristics of broadband terahertz sources. The generation of THz waves using chirped lasers has been reported [22,23]; however, there is currently a lack of investigations regarding the influence of laser spot size on the property of the THz wave in a PFT system. In this paper, the chirped property of THz waves generated in an LN prism using the PFT method is discussed. The THz spectra were investigated for three different pump-beam spot sizes. A scanning technology was employed using a slit to investigate the chirped property of the THz pulse. In addition, the THz output power was studied under the same pump power density.

## 2. Experimental Setup

**Figure 1:**
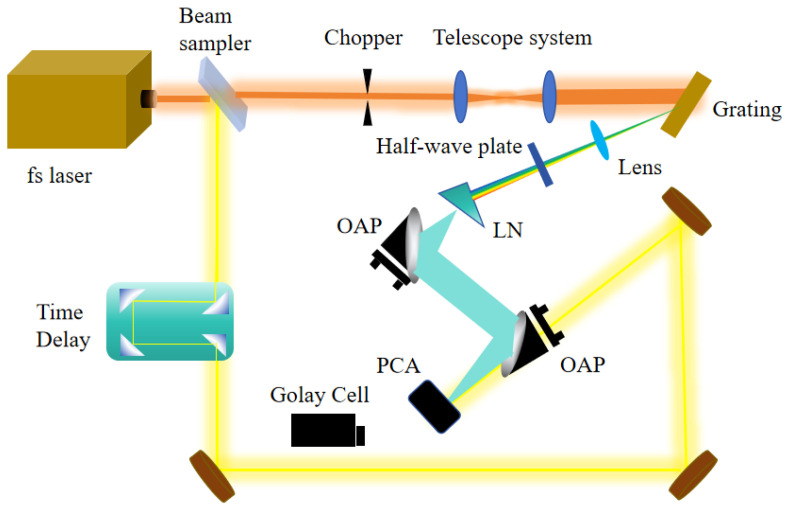
Experimental setup for THz generation in LN using the PFT technique.

The experimental configuration is depicted in Figure 1. Pump pulses were generated using an Yb-PCF amplifier with a central wavelength of λ = 1040 nm and a pulse width of τFWHM ≈ 115 fs, delivering up to 0.05 µJ of energy at a repetition rate of 43 MHz. The nonlinear crystal was a c-cut stoichiometric LiNbO_3_ (LN) crystal doped with 0.5% MgO (Eachwave scientific instrument Co., Ltd, Shanghai, China), possessing the shape of a right triangle prism with a base angle of 64°. The crystal thickness was 5 mm, the hypotenuse length was 11 mm, and the arm length was 5 mm. The pump spot size could be adjusted using a telescope system. A grating with a groove density of 1200 lines/mm at an incident angle of −22° was used to create the PFT. A half-wave plate was used to adjust the polarization of the incident laser beam parallel to the optical axis of the LN crystal. The PFT pulses were focused into the LN prism via a lens positioned behind the grating with a focal length of 60 mm. The THz radiation was collected and focused onto the photoconductive antenna (PCA) (Eachwave scientific instrument Co., Ltd, Shanghai, China) or Golay cell (Tydex, GC-1P, Sanit Peterburg, Leningrad Region, Russia) using two 90° off-axis parabolic (OAP) mirrors with a focal length of 45 mm to record the THz pulse and measure the THz power. Additionally, the THz radiation was modulated at a frequency of 1000 Hz for recording the THz pulses and 10 Hz for measuring the THz output power, utilizing an optical chopper. The mirrors reflecting the pump beam were silver-coated, and the OAP mirrors were coated with gold film.

## 3. Experimental Results and Discussion

**Figure 2:**
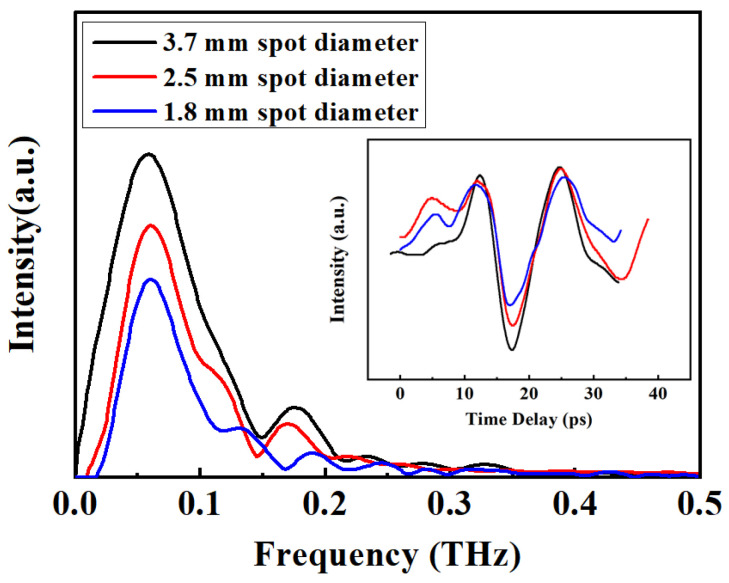
Spectrum and temporal waveforms of the THz pulse measured using a photoconductive antenna.

The spectrum and temporal waveforms of the THz pulse generated under the three studied pump spot sizes for the same pump power intensity are illustrated in Figure 2. The spectral width increased with the enlargement of the laser spot size. The angular dispersion induced by the grating is approximately equal for the pump beam with three different spot sizes when the incidence angle on the grating remains constant. The pump beam with a large spot size contains a multitude of frequency components. The majority of these frequency components overlap in the pump pulse, resulting in the generation of a chirp-free pulse. This leads to an enhanced utilization (even for the edge frequency of the diffracted beam) of the laser beam responsible for terahertz wave generation. The pump beam with a smaller spot size contains fewer frequency components, and the overlapping components will be reduced after dispersion. The separated frequency components that do not overlap are ineffective for the generation of the THz wave. This implies that the edge frequency components of the diffracted beam cannot effectively participate in optical rectification for THz wave generation, leading to a slightly narrower THz spectrum.

**Figure 3:**
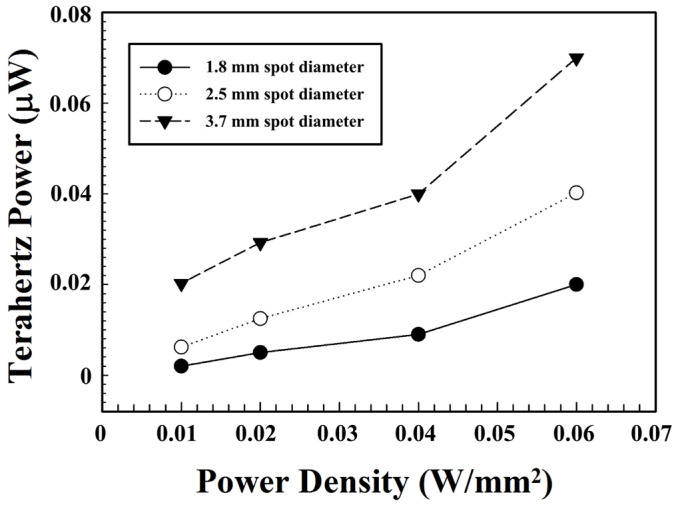
Dependence of THz power on the pump power density.

The relationship between the generated THz output power and the pump power intensity is illustrated in Figure 3 for the pump beam with three different spot sizes. The THz output power increased with the pump spot size under the same pump power density. The maximum THz power generated for the pump with a spot diameter of 3.7 mm was about three times larger than that of the 1.8 mm diameter spot, while maintaining the same pump power density. This result may have been caused by the angular dispersion of the laser beam. For the pump beam with a small spot size, the diffracted beams of different frequencies cannot be superimposed together. Therefore, the edges of the diffracted beam cannot be utilized for THz wave generation. For the pump beam with a large spot size, under the same angle dispersion, different-frequency diffracted beams would be superposed even at the edge sides. Consequently, higher THz output power can be achieved.

The PFT angle may undergo slight variations due to changes in the pump spot size. To ensure that the PFT angle remains within an acceptable range for phase matching, a study on the relationship between PFT angle and spot diameter was conducted using the ray-tracing method. The two edges’ wavelengths of the diffraction beam were 1010 nm and 1070 nm, respectively, and the central wavelength was 1040 nm. The diffraction angle of the three beams can be obtained using the grating equation $\sin\theta =\frac{\lambda }{d}-\sin\alpha$. The optical path difference between 1070 nm and 1010 nm of the diffraction beam is shown as $s_{1}=\frac{b_{0}}{\cos\theta_{2}}-\frac{b_{0}}{\cos\theta_{1}}$, where *θ*_1_ and *θ*_2_ are the diffraction angles of the 1070 nm and 1010 nm laser, and *b*_0_ is the spot diameter. The optical path difference caused by different spot sizes is expressed as $s_{2}=d_{AB}\cdot \cos\left(\frac{\pi }{2}-\alpha \right)$, where *d_AB_* is the spot area of the diffraction beam by the grating, expressed as $d_{AB}=\left(\tan\theta_{1}-\tan\theta_{2}\right)\cdot b_{0}$. The optical path difference *s*_3_ is expressed as $s_{3}=\frac{c\cdot t}{n_{1}}-\frac{c\cdot t}{n_{2}}$, where *n*_0_, *n*_1_, and *n*_2_ indicate the linear refractive index at 1040 nm, 1070 nm, and 1010 nm; *t* is the travel time of the pump laser in the crystal, expressed as $t=\frac{l\times n_{0}}{c}$; and *l* is the propagation path in the crystal. The equivalent beam length in the propagation direction can be expressed as $h=d_{AB}\cdot \sin\left(\frac{\pi }{2}-\alpha \right)$. Finally, the PFT angle can be expressed as $\tan\gamma =\frac{h}{s_{1}+s_{2}+s_{3}}=\frac{(\tan\theta_{1}-\tan\theta_{2})\cdot b_{0}\cdot \cos\alpha }{\frac{b_{0}}{\cos\theta_{2}}-\frac{b_{0}}{\cos\theta_{1}}+(\tan\theta_{1}-\tan\theta_{2})\cdot b_{0}\cdot \sin\alpha +l\left(\frac{n_{0}}{n_{1}}-\frac{n_{0}}{n_{2}}\right)}$. The PFT angle for the pump beam with three different spot sizes is calculated as 64.91°, 64.16°, and 63.42°, respectively. Consequently, this slight variation in angle is unlikely to have a significant impact on the THz output power.

**Figure 4:**
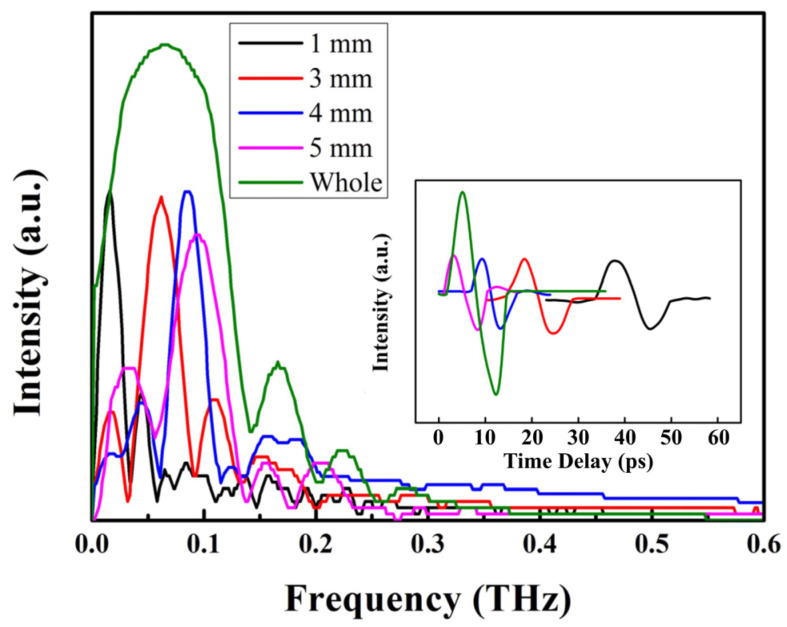
Scanning spectrum of the THz pulse for the pump beam with a spot diameter of 1.8 mm.

**Figure 5:**
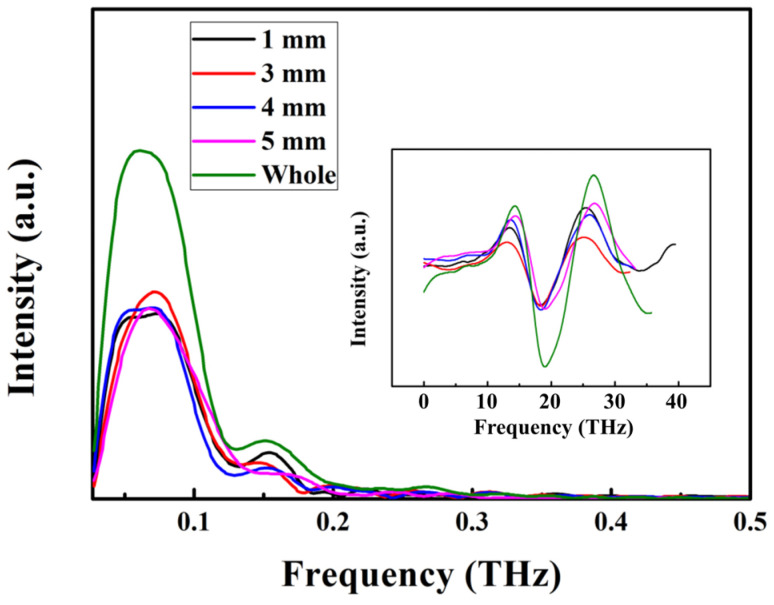
Scanning spectrum of the THz pulse for the pump beam with a spot diameter of 3.7 mm.

The chirped property of the THz wave was investigated for the pump beam with a spot diameter of 1.8 mm and 3.7 mm using spectrum scanning technology. The resulting scanning spectra of the THz pulses are presented in Figure 4 and Figure 5. In our experiment, a 1 mm wide slit (with an absorption magnitude of 80% for THz waves) was employed. Initially, the slit was positioned on the left side of the LN crystal. Subsequently, as the slit was incrementally moved to the right by a step distance of 1 mm each time, THz spectra were recorded. For the pump beam with a spot diameter of 3.7 mm, each scanning spectrum obtained at a step distance of 1 mm exhibited an equivalent THz spectral width compared to that observed without using a slit, differing only in terms of intensity. For the pump beam with a spot diameter of 3.7 mm, the different-frequency components of the pump beam were overlapped before THz generation, resulting in a nearly chirp-free THz spectrum. However, for the pump beam with a spot diameter of 1.8 mm, the different-frequency components of the pump beam failed to overlap, leading to the generation of a chirped THz wave. So, the scanning spectrum with a slit exhibited a narrower THz spectral width compared to that without a slit. Additionally, the central frequency of the scanning spectrum increased due to the frequency distribution caused by angular dispersion.

## 4. Conclusions

In summary, we have systematically studied the effect of pump-beam spot size on THz generation in an LN crystal based on PFT technology. A chirped laser beam with grating was employed with a spot diameter of either 1.8 mm, 2.5 mm, or 3.7 mm for THz wave generation. The THz spectra were recorded, and a wider THz spectrum width was obtained when the pump spot diameter was 3.7 mm. The maximum THz power generated for the pump beam with a spot diameter of 3.7 mm was about three times larger than that of the 1.8 mm diameter spot, while maintaining the same pump power density. The scanning spectrum of the THz range was also investigated for the pump beam with spot diameters of 1.8 mm and 3.7 mm. The results demonstrate a pronounced chirped characteristic in the THz pulse when using a smaller spot diameter. This research indicates that a chirped THz wave may be caused by a chirped laser beam, and the THz wave generated by a chirped pump laser in optical rectification is proven to be a mixture of various discrete frequencies.

## Data Availability

The raw data supporting the conclusions of this article can be made available by the authors on request.
